# Advanced hydrogel-based drug delivery systems for psoriasis management: From material design to multi-target therapies

**DOI:** 10.1016/j.ijpx.2026.100599

**Published:** 2026-07-04

**Authors:** Huijuan Liang, Huina Cao, Shulin Pu, Runqi Xu, YaQi Xu, Chengxiao Wang

**Affiliations:** School of Life Science and Technology, Kunming University of Science and Technology, Kunming 650500, China

**Keywords:** Psoriasis, Hydrogels, Transdermal drug delivery, Stimuli-responsive, Multi-target therapies

## Abstract

Psoriasis is a chronic inflammatory skin disorder driven by the interplay of genetic, immunological, and environmental factors. Hydrogels, featuring excellent biocompatibility and tunable structures, have emerged as pivotal platforms for topical psoriasis management. Although existing studies have explored hydrogel drug delivery and sustained release, they often remain confined to single-target interventions or basic formulations. This review updates recent advances by elucidating intervention mechanisms from the pathological microenvironment. We highlight synergistic strategies using smart hydrogels and nanogels, with emphasis on their core strengths in sustained/controlled release, enhanced penetration, and dosage form design. Finally, future perspectives and targeted strategies are proposed to address critical bottlenecks in clinical translation, including manufacturing standards and long-term safety. This work aims to provide theoretical guidance for developing next-generation hydrogel formulations with high efficacy and low toxicity.

## Graphical abstract

**Figure:**
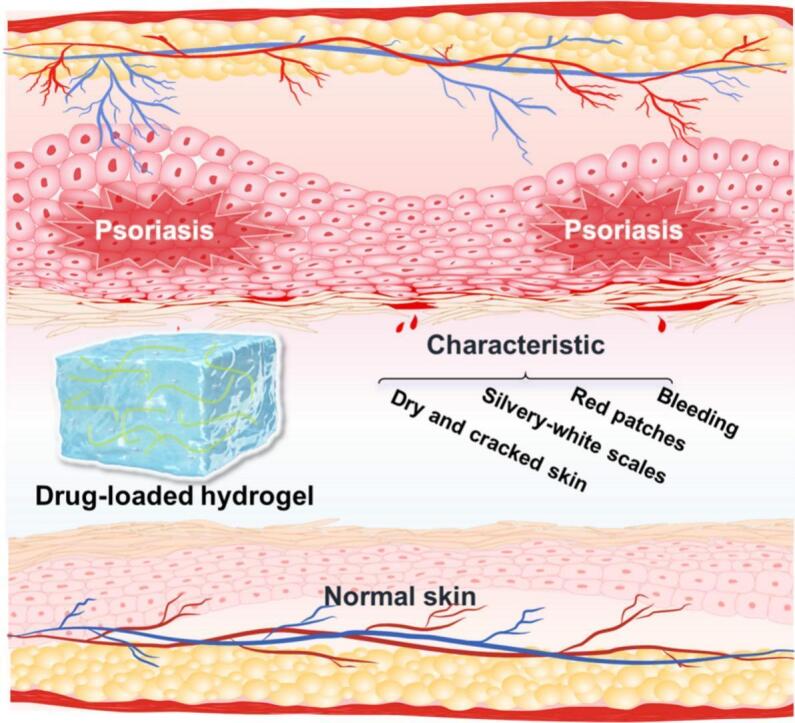

## 1 Introduction

**Fig. 1:**
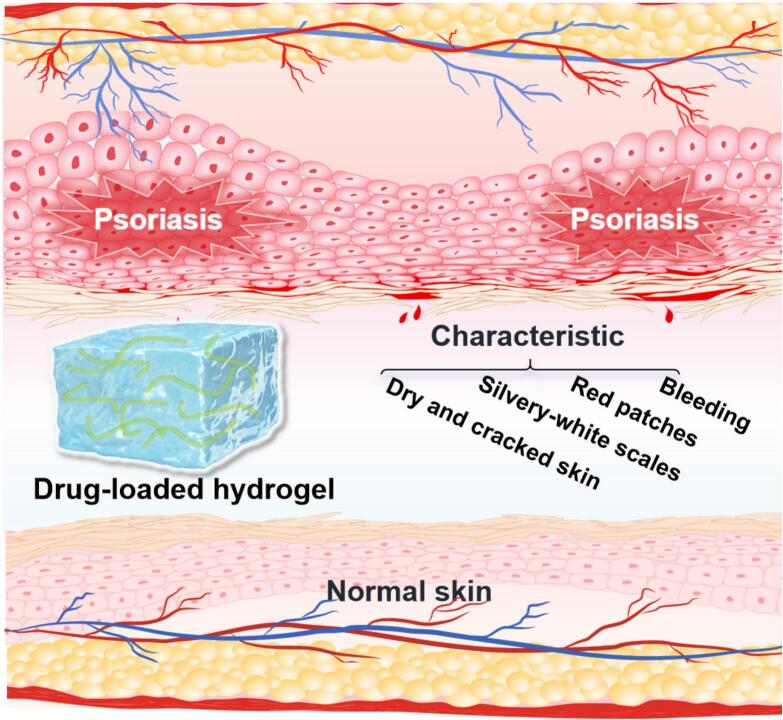
Architecture of a psoriatic lesion (top) and a non-psoriatic lesion (bottom).

Psoriasis is a chronic relapsing inflammatory skin disease with a global prevalence of 2%–3%, affecting over 125 million people and causing a heavy socioeconomic burden (Damiani et al., 2021; Xie et al., 2021) (Fig. 1). The typical clinical presentation is persistent erythema, silvery-white scales, and severe pruritus. The pathogenesis of the disease is complex and involves genetic predisposition versus metabolic imbalance, with Interleukin-23 (IL-23)/T helper 17 cell (Th17) axis-mediated immune dysregulation and impaired epidermal barrier at its core (Koo et al., 2017; Yuan et al., 2019).

The local treatment of psoriasis still faces multiple challenges, such as insufficient drug penetration, systemic side effects and low patient compliance. Traditional semi-solid formulations (e.g., tacrolimus (TAC) ointment) are difficult to reach the deep epidermis and dermis due to the viscous matrix and poor permeability, while systemic drugs (e.g., cyclosporine A (CSA)) have a faster onset of action; however, they are associated with hepatorenal toxicity and immunosuppression, limiting long-term use (Wei et al., 2026). Therefore, hydrogel-based drug delivery systems (HDDS) with high water content have received extensive attention due to their excellent biocompatibility and controlled release ability (Su et al., 2023a).

HDDS can serve as a versatile drug carrier platform to precisely design release kinetics by modulating polymer network structure and cross-linking density to maintain effective therapeutic concentrations at the lesion site and extend dosing intervals (Dai et al., 2023). The introduction of nanotechnology can further facilitate drug penetration into the stratum corneum (SC) and target deep tissues. In particular, smart responsive hydrogels (temperature, pH, and ROS responsive) enable synchronous precision release with the psoriatic microenvironment (Thakur et al., 2023), improving efficacy while reducing systemic exposure (Mitra et al., 2025).

Although recent reviews have explored pathogenesis (Cao et al., 2024), polymer material selection (Vasowala et al., 2024), or single stimuli-responsive mechanisms, a systematic framework to integrate these elements is lacking. Different from the existing literature, this review starts from material selection and cross-linking mechanism, and extends to ionic liquid (IL) gels, eutectogel, and spray/microneedle integration systems, focusing on the elucidation of hydrogel-mediated multidimensional synergistic regulation strategies (Shinde et al., 2025; Zhu et al., 2025). Finally, we review the current technical bottlenecks and future development, aiming to provide new perspectives for the precise local treatment of psoriasis.

Schematic illustration depicting the pathological features of psoriasis vulgaris compared to normal skin architecture. The upper panel highlights characteristic manifestations of psoriatic lesions, including erythema, silvery-white scales, bleeding upon scale removal, and dry/cracked skin. The central panel illustrates a drug-loaded hydrogel system, which serves as a topical therapeutic intervention. The lower panel represents healthy skin.

## 2 Overview of pathogenesis

Psoriasis is a chronic, immune-mediated inflammatory dermatosis arising from the interplay between genetic susceptibility and environmental triggers (Chen and Tsai, 2018; Costanzo et al., 2018). The disease is pathologically characterized by excessive keratinocyte proliferation and impaired differentiation, which are driven by a dysregulated cutaneous and systemic immune network (Wang and Lai, 2024; Zhou et al., 2022).

### 2.1 Genetic susceptibility

Genetic predisposition is a fundamental determinant of psoriasis onset and progression. Among the identified susceptibility loci, psoriasis susceptibility 1 (PSORS1), located within the human leukocyte antigen class I cw6 (HLA-Cw6) region on chromosome 6p21.3, represents the most prominent genetic association. This locus is believed to influence antigen presentation and to contribute to the aberrant activation of the: nuclear factor kappa-light-chain-enhancer of activated B cells (NF-κB) and signal transducer and activator of transcription 3 (STAT3) signaling pathways, thereby promoting inflammatory responses in psoriatic skin (Fan et al., 2008).

### 2.2 Environmental triggers

Environmental triggers such as streptococcal infection and skin trauma can disrupt the epidermal barrier and activate plasmacytoid dendritic cells (DC) to produce IL-23, which promotes Th17 and type 17 CD8^+^ T (Tc17) cells responses and activates the IL-23/IL-17 axis (Bai and Srinivasan, 2016; Yadav et al., 2020). Cytokines including interleukin-17 A (IL-17 A), interleukin-22 (IL-22), and tumor necrosis factor-alpha (TNF-α) accelerate epidermal turnover from about 28 days to 3–4 days, leading to erythema (Kanda, 2021).

**Fig. 2:**
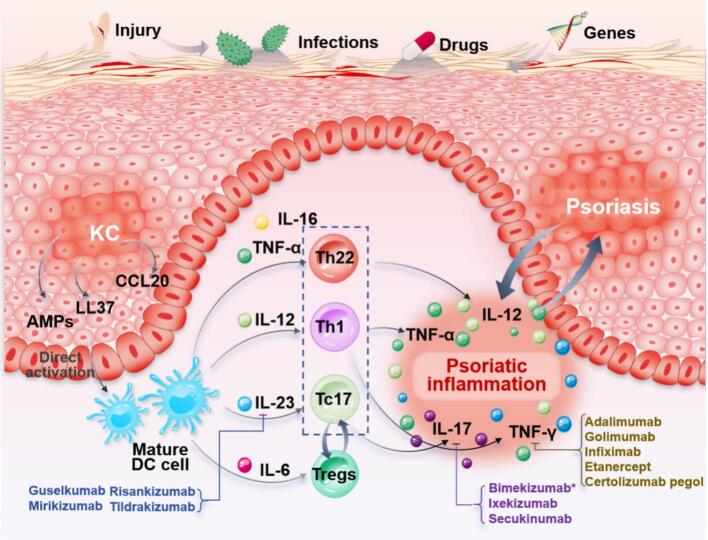
Pathogenesis of psoriasis.

Other factors that promote inflammation include lipid metabolism disorders, ROS, exosomal miRNAs such as miR-381-3p and metabolic reprogramming with glucose transporter 1 overexpression and lactate accumulation (Jiang et al., 2021). Disease persistence is also maintained by tissue-resident memory T cells (Singh et al., 2022) (Fig. 2).

**Fig. 3:**
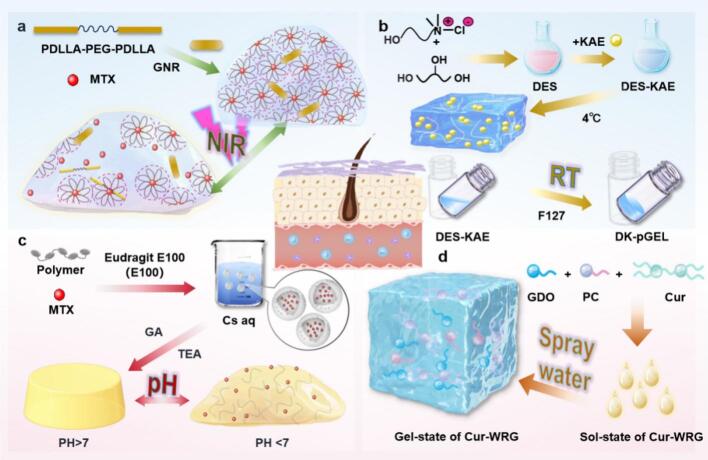
treatment of psoriasis with responsive hydrogels. (a) Photothermal response: Thermosensitive hydrogels were prepared using PDLLA-PEG-PDLLA combined with GNRs encapsulating MTX. Reprinted from (Chen et al., 2025a), with permission from Wiley. (b) Temperature response: The hydrogel exists as an injectable sol below 25 °C and undergoes a sol–gel transition upon contact with skin. Reprinted from (Su et al., 2023a), with permission from American Chemical Society. (c) pH response: A pH-dependent nanocarrier system encapsulates MTX in pH-sensitive Eudragit E100 polymeric nanoparticles and is loaded into hydrogels. Reprinted from (Asad et al., 2021), with permission from MDPI. (d) Water response: A “water-triggered” smart gel was prepared, whose viscosity increases rapidly after water spraying. Reprinted from (Yao et al., 2023), with permission from Elsevier.

**Fig. 4:**
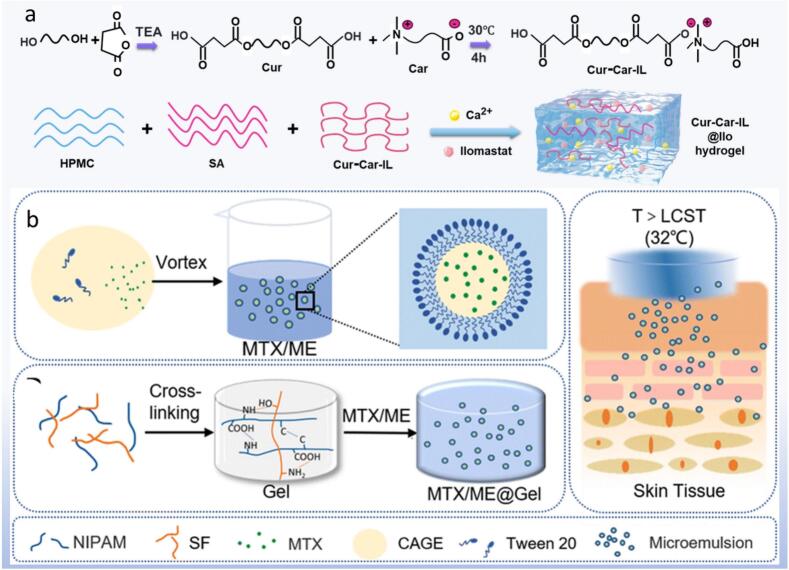
IL hydrogels. (a) A physically crosslinked pH-responsive IL hydrogel Cur-Car-IL was synthesized by reacting CUR succinic acid with carnitine and then mixing with SA and HPMC. Reprinted from (Lu et al., 2024a), with permission from American Chemical Society. (b) A thermos-responsive IL hydrogel prepared using choline and glycine betaine eutectic solvent (CAGE) for transdermal delivery of MTX. Reprinted from (Shu et al., 2023), with permission from The Royal Society of Chemistry.

**Fig. 5:**
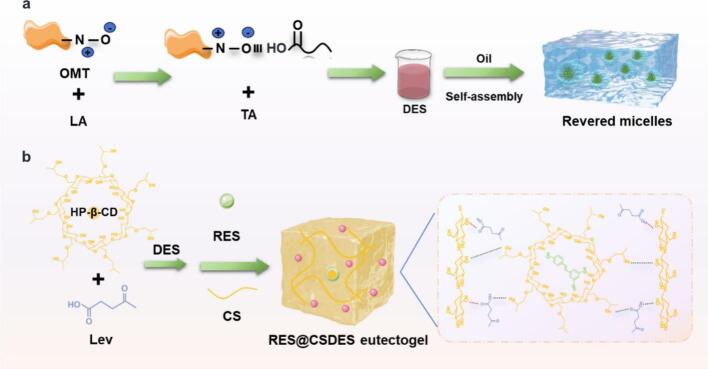
Selective preparation of hydrogels with novel materials. (a) Self-assembled reverse micelle system. A surfactant-free and water-free DES reverse micelle system was constructed using natural precursors OMT and LA. Reprinted from (Li et al., 2024a), with permission from BioMed Central. (b) Eutectogels. By constructing supramolecular cyclodextrin-DES enhanced CS system, green cross-linking was achieved by hydrogen bonding and electrostatic interaction, and eutectic gels were prepared for transdermal delivery of RES. Reprinted from (Li et al., 2025), with permission from Elsevier.

**Fig. 6:**
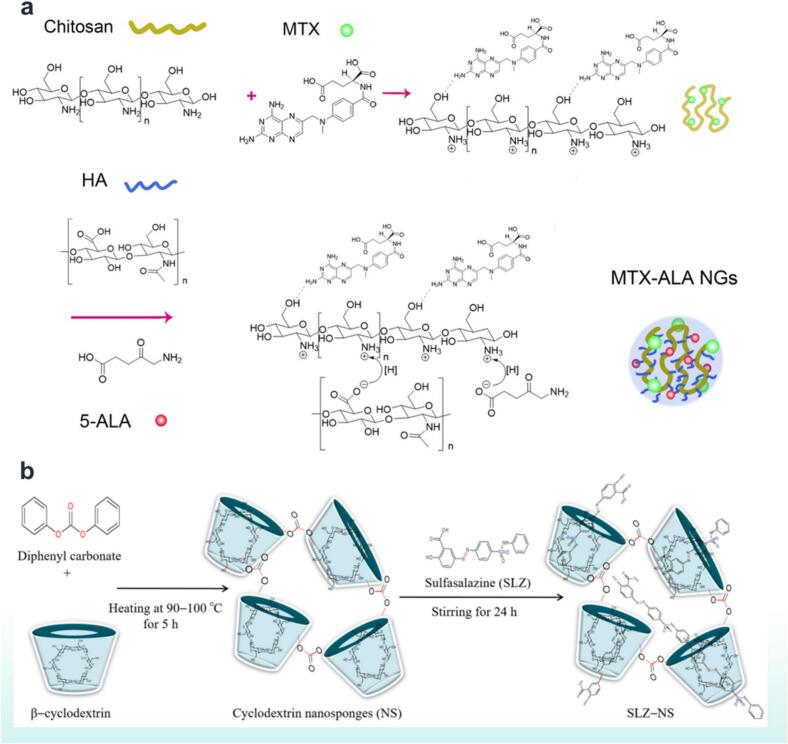
Nano-hydrogels (a) Nanoparticle hydrogels. Preparation of CS/HA nanogels by electrostatic self-assembly method. Reprinted from (Wang et al., 2022), with permission from American Chemical Society. (b) Nano-sponge hydrogel. Fabrication of sulfasalazine nano-sponges using diphenyl carbonate as a crosslinking agent. Reprinted from (Kumar et al., 2025), with permission from MDPI.

**Fig. 7:**
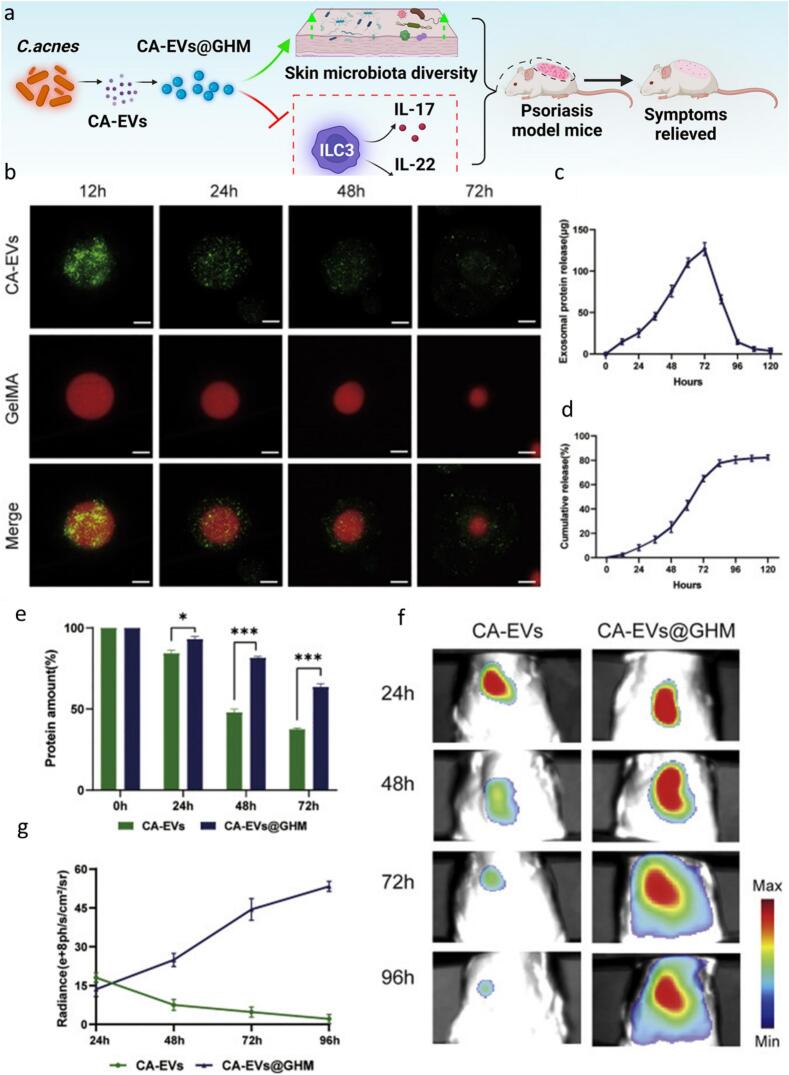
CA-EVS@GHM modulates skin microecology and restores skin barrier function in psoriatic mice. (a) Mechanism schematic: CA-EVs@GHM ameliorates psoriasis-like symptoms by remodeling the skin microbiota to inhibit IL-17 and IL-22 secretion by ILC3 cells. (b) Degradation of CA-EVs@GHM under 37 °C for 12 h, 24 h, 48 h and 72 h. (c) Quantitative analysis of exosomal protein released from CA-EVs@GHM via BCA assay. (d) Cumulative release of exosomal protein from CA-EVs@GHM via BCA assay. (e) Total protein of CA-EVs@GHM and CA-EVs after incubation at 37 °C. (f) In Vivo fluorescence imaging of live animals at different time points following subcutaneous injection of Dio-labeled CA-EVs@GHM and CA-EVs. (g) Quantitative analysis of CA-EVs retention in vivo through radiance intensity measurement. Reprinted from (Xu et al., 2024), with permission from Elsevier.

**Fig. 8:**
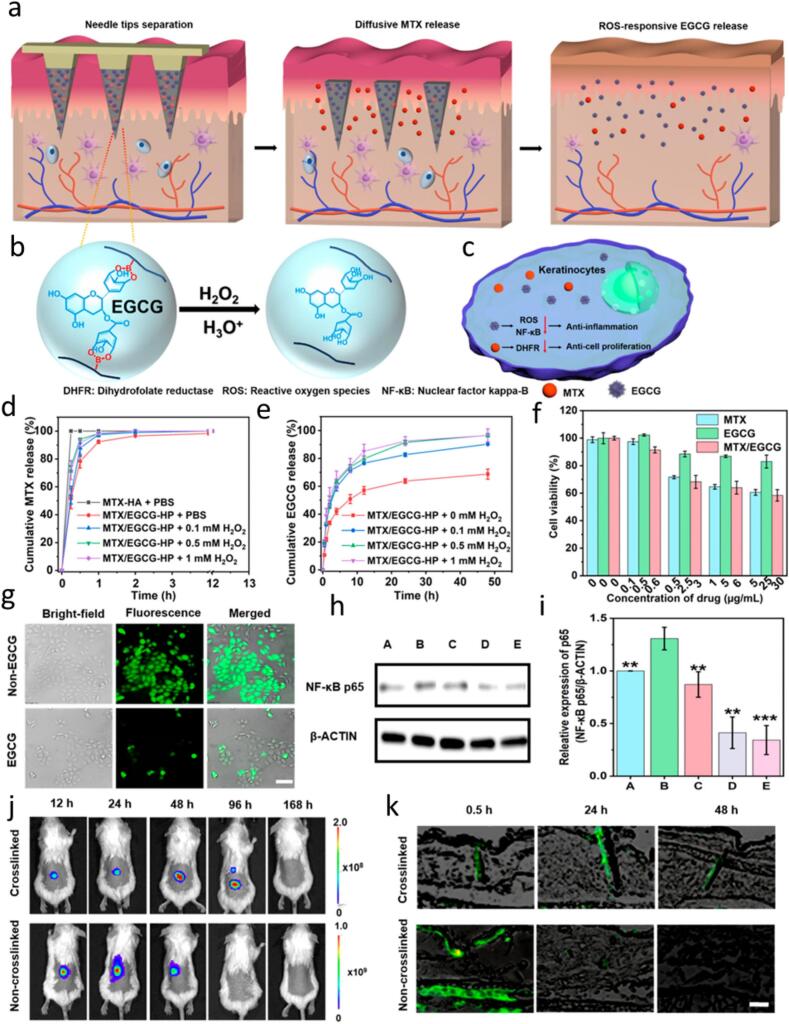
ROS-responsive drug release behavior of EGCG–HP gel-based microneedles. (a) MN tips separate with the supporting arrays to stay in skins. (b) Sustained release of EGCG is triggered by H2O2 in the psoriatic skins. (c) EGCG prohibits the NF-κB inflammatory pathways activation by scavenging ROS. (d) MTX and (e) EGCG from the MN system at different concentrations of H2O2. (f) Cell viability of HaCaT cells at various concentrations of MTX, EGCG, and MTX/EGCG. (g) Bright-field, fluorescent, and merged images of HaCaT cells (H_2_O_2_ treated) with or without incubation of EGCG via a DCFH-DA assay. (h) Protein expression of NF-κB p65 and β-ACTIN as evaluated by Western blot analysis. (i) Densitometry data generated from the Western blots of HaCaT cells under different conditions. (j) Representative in vivo fluorescent images of mice at different time points after insertion of cross-linked EGCG-HP gel or non-cross-linked HA dissolving MN patches into the psoriatic skin of mice. (k) The cross-linked HA MN had better penetration effect than the other non-cross-linked HA dissolving MN to the mice. Reprinted from (Bi et al., 2023), with permission from ACS.

**Fig. 9:**
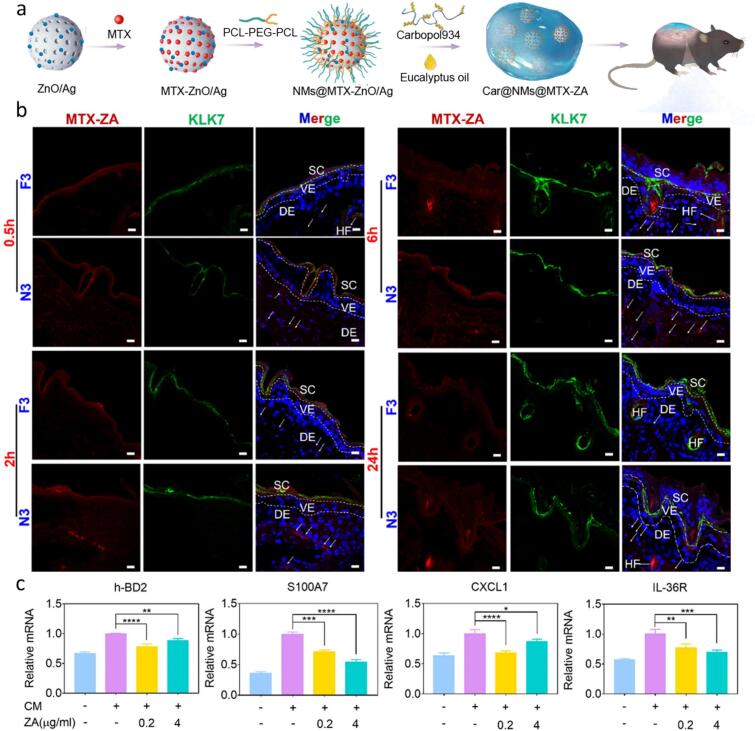
Schematic illustration and therapeutic mechanism of the nanocomposite hydrogel (Car@NMs@MTX-ZA). (a) Schematic diagram showing the fabrication process of the Car@NMs@MTX-ZA hydrogel. (b) Photograph and histopathological images of mice back skin in skin irritation study. (c) Relative mRNA expression of pro-inflammatory markers in HaCaT cells was analyzed by quantitative PCR (qPCR). Reprinted from (Xu et al., 2022), with permission from Elsevier.

**Fig. 10:**
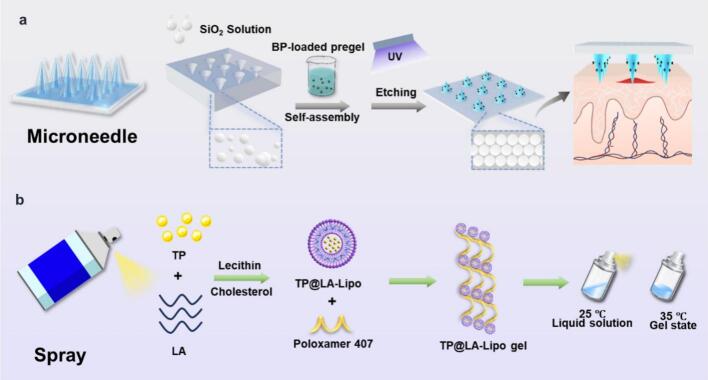
Advanced hydrogel formulations. (a) HMNS. The thermal response framework was constructed by colloidal crystal template method, and the obtained HMNS could be used as thermal response scaffolds, and the controlled drug release was achieved by NIR trigger. Reprinted from (Lu et al., 2024b), with permission from Elsevier. (b) Sprayable hydrogels. A spray-on thermosensitive liposome hydrogel that forms a flexible membrane within 3–5 s of contact with the skin. Reprinted from (Shen et al., 2025), with permission from MDPI.

### 2.3 Current treatment

Current therapies include topical agents, systemic small molecules, and biologics (Han et al., 2025; Liu et al., 2024). Topical drugs include vitamin D3 analogues such as calcitriol and calcineurin inhibitors such as TAC (Fereig et al., 2021). Systemic agents include CSA (Gallo et al., 2022), Acitretin (Chen et al., 2021), and Apremilast (Chimenti et al., 2015). Biologics target TNF-α (Kirwin et al., 2025), IL-12/23 (Qi et al., 2025), IL-17 (Wang et al., 2025a), and IL-23 (Bubna and Viplav, 2024). Some pharmaceutical preparations are shown in Table 1 below.

**Table 1:** Conventional preparations for the treatment of psoriasis.

Representative Brand Name	Drug	Dosage Form	Characteristics	Manufacturer
Dovobet/Daivobet	Calcipotriol (CPT) /Betamethasone Dipropionate	Ointment, Gelatin, Cream, Foam	Synergistic effect; rapid and sustained onset. Fixed ratio cannot be adjusted individually; retains side effects of both components.	LEO Pharma
Enfu Cream	Clobetasol propionate (CP)	Cream/Ointment/Foam	Potent anti-inflammatory; anti-allergic and antipruritic effects with rapid onset. Controls only epidermal symptoms; long-term use easily leads to skin atrophy; telangiectasia and secondary infection.	Guangdong China Resources Shunfeng Pharmaceutical Co., Ltd.
Diwei	All-trans retinoic acid (TRA)	Cream	Suitable for maintenance therapy or rotation on trunk/limbs; non-steroidal. Slow onset; common local irritation (burning, peeling); photosensitive.	Shanghai Modern Pharmaceutical Co., Ltd.
Daivonex/Dovonex	CPT	Ointment/Cream/Solution	Non-steroidal; regulates keratinocyte differentiation; suitable for long-term maintenance. Slow onset as monotherapy; local irritation possible; risk of hypercalcemia with large-area use.	LEO Pharma
Protopic	TAC	Ointment	No risk of skin atrophy; suitable for face and intertriginous areas. Slower onset; local burning sensation; efficacy weaker than steroids.	Astellas Pharma Inc.
Betnovate	Betamethasone Valerate	Cream, Ointment, Lotion	Safer profile for short-term standardized use; suitable for short-term control of larger areas on trunk/limbs. Long-term or extensive use can cause skin atrophy and HPA axis suppression.	GSK (GlaxoSmithKline)
Lewei	Tazarotene (TZT)	Cream	Corrects abnormal keratinization. Strong irritation; photosensitivity.	Chongqing Huapont Pharm Co., Ltd.
Baohusan	Hydrocortisone (HCT)	Cream	Anti-inflammatory; anti-allergic and antipruritic effects. Long-term use may cause skin thinning; striae and pigment changes.	Hubei Ketian Pharmaceutical Co., Ltd.
Ixekizumab	IL-17 A inhibitor (mAb)	Solution for SC injection	Marketed	UNCOVER program
Bimekizumab	IL-17 A/F inhibitor (mAb)	Solution for subcutaneous injection	Marketed	Head-to-head vs. Secukinumab
ZL-1102	Anti-IL-17 A nanobody	Topical hydrogel	Phase 1b	First-in-class topical biologic

Although these treatments have improved disease control, they remain limited by systemic toxicity, adherence issues, and formulation constraints. Biologics are not suitable for topical use in local lesions (Pan et al., 2025).

Schematic overview of the pathogenesis of psoriasis and the corresponding therapeutic targets. The diagram illustrates how external triggers (e.g., injury, infections, drugs) lead to the activation of SC and the subsequent release of antimicrobial peptides like cathelicidin antimicrobial peptide LL-37 (LL-37) and chemokines (CCL20). This initiates an immune cascade involving mature DC, T helper 1 (Th1), T helper 22 (Th22), and Tc17 cells. The central role of the IL-23/Th17 axis is highlighted, showing the production of key pro-inflammatory cytokines (IL-17, IL-22, TNF-α). On the right, representative biologic drugs targeting this pathway (e.g., anti-IL-17, anti-IL-23, anti-TNF-α agents) are listed.

## 3 Hydrogel design

### 3.1 Overview

Psoriasis is a chronic inflammatory dermatosis characterized by aberrant keratinocyte hyperproliferation and sustained activation of the IL-23/IL-17 axis (Dascălu et al., 2024). Current clinical management relies heavily on topical agents, as summarized in Table 1. However, these conventional formulations are frequently hampered by short residence times and cumulative local irritation. To address these limitations, novel localized delivery systems have been progressively developed, as illustrated in Table 2. Collectively, therapeutic strategies for psoriasis are evolving from traditional topical vehicles toward intelligent hydrogel-based delivery platforms. By synergizing material engineering with pharmacological mechanisms, these advanced systems achieve sustained release, enhanced permeation, and precise microenvironment modulation, thereby establishing a robust foundation for innovative and effective localized therapies. Detailed discussions regarding specific therapeutic advantages are presented in Section 5.

**Table 2:** Preparation of hydrogels for the treatment of psoriasis.

Material selection	Cross-linking strategies	Drugs	Advantages	Multi-target therapies	Remarks	References
Carbomer 940; Glycerol; Ethylenediaminetetraacetic acid; Ethyl p-hydroxybenzoic acid	Physical crosslinking	TRA; Betamethasone (BD)	Enhance skin permeability and drug stability; reduce photosensitivity and irritation of TRA.	TRA regulates keratinocyte differentiation and exerts anti-inflammatory effects; BD provides potent anti-inflammation and immunosuppression.	Liposomal	(Wang et al., 2020)
Carbopol 934	Physical crosslinking	CP	Improve the solubility and stability of CP and reduce side effects; enhance the skin permeability and residence time of the drug.	Anti-inflammatory; anti-proliferative and immunomodulatory effects.	Nano sponge	(Kumar et al., 2021)
Hyaluronic acid (HA); Mxene (Ti3C2); Photothermal material	Physical crosslinking	IL-17 monoclonal antibody (IL-17mAbs)	Reduce system side effects; light-controlled release; good biocompatibility; fast skin recovery	IL-17mAbs neutralizes IL-17 cytokines to block inflammatory signaling; photothermal stimulation enhances localized release.	Microneedle	(Wu et al., 2022)
Methoxy polyethylene glycol-thioether-thiol	Self-assembly	CPT	Enhance the concentration of drugs in the lesion site; nanometer particle size; promote skin penetration; good biocompatibility.	Calcipotriol inhibits abnormal keratinocyte proliferation and differentiation; mPEG-SS scavenges ROS.	Nano-micelles	(Hua et al., 2022)
Carboxymethyl Cellulose; Sorbitan Monostearate; Polycaprolactone Nanoparticles	Physical crosslinking	HCT	Small particle size (110.5 nm) and high zeta potential (−58.7 mV) ensure stability and dermal retention. Sustained release over 24 h reduces systemic absorption and dermal toxicity.	HCT binds to Glucocorticoid and Mineralocorticoid receptors, suppresses NF-κB pathway. NPs-in-gel matrix prevents burst release, mitigating HPA axis suppression and skin atrophy.		(Kondiah et al., 2022b)
Carbopol 934, Nanostructured Lipid Carrier (NLC)	Physical crosslinking	TZT; CPT	High entrapment efficiency (∼91%) within the imperfect crystal lattice of NLCs. Sustained release over 72 h following Higuchi kinetics.	TZT: Activates RAR-β/γ, normalizes keratinocyte differentiation CPT: Induces terminal differentiation and immunomodulation.		(Thakur et al., 2023)
N-isopropylacrylamide; Gelatin	Chemical crosslinking; physical crosslinking	CPT	Multiple drug delivery in one insertion; high drug loading and compatibility with hydrophobic drugs; mechanical strength.	Inhibits keratinocyte hyperproliferation and IL-23/IL-17 axis signaling; photothermal-synergized immunomodulation.	Microneedle	(Lu et al., 2024b)
Isopropyl myristate; Tween-80; PEG-400; R8h3-c18	Physical-self-assembling crosslinking	Curcumin (CUR)	Two-stage permeation enhancement; lesion-selective curing; paintable and printable; long-lasting and low-stimulation.	Antioxidant; anti-inflammatory; anti-proliferative effects; synergistic delivery to achieve multi-target effects	Oligopeptide self-assembled	(Chen et al., 2025b)
Chitosan (CS); polyacrylic acid; Cerium oxide nanoparticles	Chemical crosslinking; physical double network	BD	High mechanical strength; stretchable; friction-resistant; suitable for joint parts; continuous drug release.	Rapid ROS neutralization; inhibits NF-κB/MAPK pathway activation; reduces TNF-α, interleukin-6 (IL-6), and other pro-inflammatory factors.		(Yeo and Kim, 2025)

### 3.2 Materials

According to the pathological characteristics and treatment requirements of psoriasis, this section introduces the sources, crosslinking characteristics and response modes of polymer materials (Xiao et al., 2025). Representative drug-loaded hydrogels for psoriasis treatment are summarized in Table 2. The table not only lists the preparation methods of representative hydrogels, but also the response methods and therapeutic mechanisms.

#### 3.2.1 Natural polymers

Natural polymers are commonly used to prepare hydrogels because of their biocompatibility, biodegradability (Zhao et al., 2023), and reactive groups, which match the pathological requirements of psoriatic lesions (Chen et al., 2022). Representative examples include HA, cellulose, and CS. HA offers intrinsic moisturization and CD44-mediated immunomodulatory activity, contributing to barrier support and local immunomodulation (Huang et al., 2022; Trombino et al., 2019). Cellulose-based hydrogels provide a porous, water-retentive matrix that absorbs exudate and supports lesion repair (Bao et al., 2022; Kondiah et al., 2022a). CS is notable for its antibacterial and anti-inflammatory effects (Wang et al., 2023). In addition, selected natural polymer systems can be engineered for microenvironment-responsive drug release, as discussed in Section 3.4.

#### 3.2.2 Synthetic polymers

Synthetic polymers provide higher structural stability than natural polymers (Li et al., 2021; Yeo and Kim, 2025). Polyacrylamide is valued for its high mechanical strength and water absorption, with tunable swelling and mechanical properties for use in psoriatic environments (Zhang et al., 2023b). Polyethylene glycol is widely used to construct elasticity suitable for moving skin areas such as joints and folds (Lerma et al., 2024; Zhang et al., 2023a). Carbomers rich in carboxylic acid groups swell under neutral or alkaline conditions to form binder matrices with sustained-release capabilities (Rossi et al., 2012; Zhang et al., 2020).

### 3.3 Crosslinking

Hydrogel crosslinking is classified as physical or chemical (Singh et al., 2021; Vasowala et al., 2024). Physical crosslinking relies on noncovalent interactions and typically yields reversible, self-healing (Khattak et al., 2024), making them suitable for exudate absorption and moisture retention (Valipour et al., 2023). Their relatively low mechanical strength, however, limits use to mild, superficial lesions or rapid-release applications (Devi V. K et al., 2021). For example, Pluronic F127 gels undergo in situ gelation at skin temperature, facilitating convenient administration and rapid release (Su et al., 2023a). In contrast, chemical crosslinking forms covalent networks with superior mechanical integrity and resistance to friction and enzymatic degradation. CS polyacrylic acid double-network hydrogels, for instance, improve drug retention at the lesion site and thereby enhance therapeutic efficacy (Yeo and Kim, 2025) (Table 2 and Table 3).

**Table 3:** Stimuli responsiveness of hydrogels.

Material selection	Cross-linking strategies	Drugs	Response mechanisms	Advantages	Multi-target therapies	References
Pluronic F127(20% *W*/*V*); Olive oil (5–30%); Phosphate buffer	Physical crosslinking	Quercetin (QT)	Temperature response	Enhance skin permeability; promote drug absorption; with good biocompatibility and stability.	QT exerts antioxidant and anti-inflammatory effects.	(Elmowafy et al., 2021)
Methoxy polyethylene glycol-thioether-thiol	Self-assembly	CPT	ROS	Enhance the concentration of drugs in the lesion site; nanometer particle size; promote skin penetration; good biocompatibility.	Calcipotriol inhibits abnormal keratinocyte proliferation and differentiation; mPEG-SS scavenges ROS.	(Hua et al., 2022)
N-isopropylacrylamide; silk fibroin; IL microemulsion (IL-ME)	Chemical cross-linking; physical cross-linking	MTX	Temperature response	Improve MTX solubility (9 times); enhance skin penetration (6 times); release; antibacterial activity; low toxicity.	Preparation of MTX carriers; IL-ME enhances solubility and permeability.	(Shu et al., 2023)
Phosphatidylcholine; Glycerol dioleate (GDO); Ethanol; Propylene glycol; Tween 80	Physical crosslinking	CUR	Water responsive	Easy to apply and attach; high viscosity; promote drug penetration; enhance efficacy; low toxicity; good biocompatibility.	CUR exerts anti-inflammatory, antioxidant and immunomodulatory effects; WRG enhances dermal drug retention and penetration	(Yao et al., 2023)
Phenylboronic acid modified HA; Epigallocatechin-3-gallate (EGCG)	Dynamic covalent crosslinking; Hydrogen bonding	MTX; EGCG	ROS; pH response	EGCG has natural antioxidant; anti-inflammatory; microneedles can extend the time of drug action.	MTX inhibit abnormal keratinocyte proliferation; EGCG scavenges ROS and suppresses the NF-κB inflammatory pathway.	(Bi et al., 2023)
Poloxamer 407 (P407); Poloxamer 188; PEG 6000; Snap	Physical crosslinking	Berberine; Coptisine; Phellodendrine	Temperature response	Easy to apply and locate; high skin permeability (SNAP promoter); low irritation; good biocompatibility.	Berberine: inhibits the IL-17/IL-23 pathway; Coptisine: Antioxidant; Phellodendrine: Antibacterial, inhibits keratinocyte hyperproliferation.	(Su et al., 2023b)
Pluronic F127; Glycerol; Choline chloride	Physical crosslinking of temperature sensitivity	Kaempferol	Thermosensitive phase transition	Good adhesion; sustained release; antioxidant; antiproliferative; low toxicity.	Anti-inflammatory and antioxidant effects; inhibits keratinocyte proliferation; scavenges ROS; downregulates TNF-α, IL-6, and IL-17 A.	(Su et al., 2023a)
N-isopropylacrylamide; Gelatin	Chemical crosslinking; physical crosslinking	CPT	Photothermal responsiveness	Multiple drug delivery in one insertion; high drug loading and compatibility with hydrophobic drugs; mechanical strength.	Potent local anti-inflammation; inhibits keratinocyte hyperproliferation and IL-23/IL-17 axis signaling; photothermal-synergized immunomodulation.	(Lu et al., 2024b)
CUR; Carnitine; IL; Sodium alginate (SA); Hypromellose (HPMC)	Ionic crosslinking; physical crosslinking	Ilomstat	pH response	Sustained release to maintain therapeutic concentrations; reducing systemic side effects.	Anti-inflammatory-protease inhibition synergy; ferroptosis regulation; barrier repair.	(Lu et al., 2024a)
Isopropyl myristate; Tween-80; PEG-400; R8h3-c18	Physical-self-assembling crosslinking	CUR	Enzyme response	Two-stage permeation enhancement; lesion-selective curing; paintable and printable; long-lasting and low-stimulation.	Antioxidant; anti-inflammatory; anti-proliferative effects; synergistic delivery to achieve multi-target effects	(Chen et al., 2025b)
PDLLA-PEG-PDLLA; Gold nanorods (GNRs)	Physical crosslinking of temperature sensitivity	MTX	Thermal response; photothermal response	Sprayable; skin-tight; NIR-triggered precise release; enhanced skin penetration; no skin irritation.	Photothermal therapy and chemotherapy; GNRs-induced thermal effect triggers keratinocyte apoptosis while promoting MTX release; synergistic anti-psoriatic action.	(Chen et al., 2025a)
CS; Hydroxypropyl-β-cyclodextrin (HP-Β-CD); Levulinic acid	Physical crosslinking	Resveratrol (RES)	Water-responsive	Reversible adhesion; high RES solubility (increased by 10,000 times) and stability.	RES exerts antioxidant, anti-inflammatory, and anti-proliferative effects on keratinocytes.	(Li et al., 2025)
CS; polyacrylic acid; Cerium oxide nanoparticles	Chemical crosslinking; physical double network	BD	ROS	High mechanical strength; stretchable; friction-resistant; suitable for joint parts; continuous drug release.	Rapid ROS neutralization; inhibits NF-κB/MAPK pathway activation; reduces TNF-α, IL-6, and other pro-inflammatory factors.	(Yeo and Kim, 2025)
Soy lecithin; P407; Cholesterol	Physical crosslinking	Tea polyphenols; Lactic acid (LA)	Temperature response	Easy to use; instant film-forming; sustained release and long-acting.	Antibacterial, anti-inflammatory; immune microenvironment remodeling; barrier repair.	(Shen et al., 2025)

### 3.4 Stimuli-responsive

The psoriatic microenvironment—characterized by mildly elevated temperature, slightly acidic pH, upregulated ROS, and compromised barrier function—serves as an endogenous trigger for stimuli-responsive hydrogels (Ricci et al., 2023). By integrating environmentally sensitive groups and tunable crosslinking architectures, such hydrogels enable lesion-site-specific, microenvironment-modulated drug release, as summarized in Table 3 and schematically depicted in Fig. 3.

Thermo-responsive and photothermal systems are extensively employed to modulate drug release kinetics (Wu et al., 2022). Poly (D, l-lactide) -*block*-poly (ethylene glycol) -*block*-poly (D, l-lactide) (PDLLA-PEG-PDLLA) undergoes a sol-gel transition upon near-infrared (NIR) irradiation, facilitating in situ solidification and sustained release. Complementary photothermal agents augment localized hyperthermia to trigger the on-demand release of methotrexate (MTX) (Chen et al., 2025a) (Fig. 3(a)). Additionally, deep eutectic solvent (DES)-or polyphenol-based systems exploit temperature-induced hydrogen bond rearrangement and hydrophobic interactions to reconstruct the gel network, thereby enhancing drug residence within the lesion (Su et al., 2023a) (Fig. 3(b)). In contrast, pH-responsive systems exploit the mildly acidic inflammatory microenvironment to achieve lesion-selective drug release (Asad et al., 2021) (Fig. 3(c)). Furthermore, water-responsive formulations based on phospholipids or surfactants transition from low-viscosity precursors to cohesive films upon contact with tissue exudates, ensuring rapid adhesion and stable coverage (Yao et al., 2023) (Fig. 3(d)). Building on these triggers, ROS-responsive hydrogels utilize dynamic covalent bonds or redox-active components to provide feedback regulation correlated with inflammation severity, simultaneously exerting antioxidant effects (Hua et al., 2022).

Collectively, these hydrogels integrate environmentally sensitive moieties into 3D polymeric networks, affording prolonged drug residence and precise spatiotemporal release control within psoriatic lesions (Yamanaka et al., 2021).

## 4 Advanced hydrogel design

### 4.1 Ionic liquid hydrogels

IL hydrogels are novel functional materials composed of ILs and polymer networks (ChangCunYan et al., 2023). ILs may disrupt the tight-packed lipid structure of the SC, facilitate skin penetration, and improve drug delivery through hydrogen bonds or ion-pair interactions (Sultana et al., 2021; Zhu et al., 2025). Representative examples include a CUR-based pH-responsive hydrogel with strong adhesion and enhanced transdermal CUR transport (Lu et al., 2024a), and MTX (Shu et al., 2023) loaded IL hydrogels with markedly improved permeation (Fig. 4(a, b)).

Despite these encouraging findings, the current evidence is largely limited to in vitro skin penetration studies. Long-term efficacy and safety have not been fully validated in psoriasis-like animal models. Future work should therefore emphasize the development of low-toxicity, “green” ILs to support clinical translation (Ferreira and Sarraguça, 2024).

### 4.2 Eutectogels

DESs are green solvents with low melting points, typically formed by combining a hydrogen bond donor and a hydrogen bond acceptor at a specific ratio (Xu et al., 2025). When incorporated into polymer matrices, eutectogels emerge as a promising class of gel systems with favorable mechanical properties, biocompatibility (Yang et al., 2025).

DES-based carriers markedly improve the transdermal delivery of poorly soluble actives. Surfactant-free, water-free reverse micelles assembled from natural DES components boost both drug solubility and skin permeation (Li et al., 2024a) (Fig. 5(a)). Complementarily, Pluronic-based thermos-responsive gels eliminate the need for chemical cross-linkers (Elmowafy et al., 2021). Further advancement comes from supramolecular cyclodextrin DES, where hydrogen bonding and electrostatic forces drive green cross-linking within chitosan networks to deliver RES, outperforming conventional formulations (Li et al., 2025) (Fig. 5(b)).

Despite these advantages, the potential effects of high concentrations of DES on skin microecology and impaired barrier function are not fully understood, and batch-to-batch stability during scale-up remains a key challenge (dela-Huerta-Sainz et al., 2026).

### 4.3 Nano-hydrogels

Exploiting the disrupted SC in psoriatic lesions, nanogels synergize nanoscale penetration with hydrogel-mediated retention to enhance the cutaneous delivery of poorly water-soluble antipsoriatic drugs (Han et al., 2021). Integrating the advantages of nano-carriers and semisolid matrices, they represent a promising platform for localized therapy (Li et al., 2024b; Sun et al., 2017), with representative formulations summarized in Table 4 and therapeutic benefits detailed in Section 5.

**Table 4:** Preparation and advantages of nano-hydrogels.

Preparation Materials	Drug	Crosslinking/Loading Method	Advantages	Therapeutic Mechanism	Reference
Isopropyl myristate; Tween 80; PEG 400	CUR	Nanoemulsions prepared via aqueous titration; R8H3-C18 anchored via electrostatic/hydrophobic interactions	∼16 nm deformable nanoemulsions penetrate via intercellular routes of the SC; R8H3 peptide enhances transdermal delivery and cellular uptake.	R8H3 promotes cytoplasmic release of Cur following cellular internalization and endosomal escape, exerting anti-inflammatory effects.	(Chen et al., 2025b)
Glyceryl monostearate; Carbopol Ultrez 10	TAC; Thymoquinone (THQ)	NLCs prepared via emulsion-solvent evaporation; physical dispersion	Nanoscale NLCs enhance skin penetration and establish a dermal depot effect; co-delivery enables multi-target synergistic therapy.	TAC suppresses T-cell activation and pro-inflammatory cytokine release; THQ scavenges ROS and inhibits the NF-κB pathway, reducing levels of TNF-α and IL-6.	(Alam et al., 2025)
CS; HA;	MTX; 5-Aminolevulinic Acid (ALA)	Ionic gelation via electrostatic complexation	∼140 nm size suitable for transdermal delivery; enables chemo-photodynamic synergy.	MTX inhibits keratinocyte hyperproliferation; ALA is converted to protoporphyrin IX which generates ROS to induce apoptosis of aberrant keratinocytes.	(Wang et al., 2022)
β-CD; Difunctional crosslinkers	Amphiphilic drugs	Covalent crosslinking, physical dispersion	Significantly improves solubility and photochemical stability of poorly water-soluble drugs; controllable sustained release.	Anti-psoriasis drugs (EG, corticosteroid, resveratrol) are continuously released at the target site to exert anti-inflammatory and anti-proliferative effects.	(Trotta et al., 2012)
β-CD; Dimethyl Carbonate; Carbopol 934; Guar Gum	CUR; Caffeine	physical dispersion	β-CD NS significantly enhances Cur solubility (∼15-fold) and photothermal stability; NS suitable for local retention.	Cur suppresses the NF-κB and IL-23/IL-17 axes; caffeine elevates intracellular cAMP by inhibiting phosphodiesterase.	(Iriventi et al., 2020)
β-CD; Diphenyl Carbonate; Carbopol 934	Sulfasalazine	physical dispersion	β-CD NS increases sulfasalazine solubility by ∼15-fold; covalent network protects drug and ensures sustained release (78% in 24 h).	Inhibits NF-κB nuclear translocation, blocking downstream production of TNF-α and interleukins; restores antioxidant enzyme levels.	(Kumar et al., 2025)

Current fabrication strategies for such carriers primarily fall into two categories: physical crosslinking via polyelectrolyte complexation and covalent crosslinking utilizing cyclodextrin derivatives. The former typically employs natural polysaccharides, where electrostatic complexation between protonated chitosan amino groups and hyaluronic acid carboxyl groups facilitates the in situ formation of nanogel networks (Wang et al., 2022) (Fig. 6(a)). This process eliminates the need for exogenous chemical crosslinkers, yielding biocompatible carriers whose cationic components transiently modulate tight junctions within the compromised SC. Conversely, the latter strategy employs β-cyclodextrin (β-CD) as building blocks, reacting with bifunctional crosslinkers to construct three-dimensional covalent networks with nanoscale pores—termed cyclodextrin nanosponge. This rigid architecture significantly enhances the encapsulation efficiency and stability of poorly soluble drugs, enabling prolonged lesion retention, and controlled sustained release (Kumar et al., 2025) (Fig. 6(b)).

Covalently crosslinked nanonetworks offer greater stability than self-assembled counterparts. Capitalizing on the permeability of self-assembly and the robust retention of covalent networks, this synergistic strategy expands the material design space for localized psoriasis therapy (Chivers and Smith, 2017).

## 5 Therapeutic advantages of hydrogels

The psoriatic lesion presents a unique pathological microenvironment that complicates topical drug delivery (Arora et al., 2021; Diotallevi et al., 2022). Recent advances in material design and formulation engineering have yielded innovative hydrogel-based dosage forms featuring sustained delivery, stimuli responsiveness, and enhanced penetration, thereby significantly improving bioavailability and therapeutic specificity (Bharti et al., 2025).

### 5.1 Sustained release

Psoriasis presents a chronic-inflammatory microenvironment. It demands sustained local drug levels. However, free proteins and peptides undergo rapid clearance (Liu et al., 2021). As discussed above, hydrogel networks engineered via specific cross-linking strategies effectively address this limitation. This approach achieves prolonged release and reduces irritation caused by frequent dosing (Chen et al., 2023).

To address the issue of rapid clearance, cutibacterium acnes-derived extracellular vesicles (CA-EVs) were encapsulated within gelatin methacryloyl (GelMA) hydrogel microspheres (CA-EVs@GHM), sustaining a high local concentration within the skin for up to 96 h. It can also intervene in multiple nodes by regulating immune receptors, such as dysbacteriosis, excessive inflammation, and barrier repair (Xu et al., 2024) (Fig. 7). Moreover, specific probiotic strains, such as bifidobacterium breve, are able to reshape the Th17/Treg balance, reflecting the advantage of microbiota regulation in multi-target therapy (Lu et al., 2021).

CA-EVs@GHM achieve sustained EV release. This system inhibits the pathogenic ILC2-to-ILC3 transition and suppresses IL-17 A/IL-22 secretion. Concurrently, it restores skin microbiota diversity, inhibits S. aureuscolonization, and upregulates barrier protein expression to promote keratinocyte repair, thereby alleviating psoriatic inflammation.

### 5.2 Controlled release

Stimuli-responsive hydrogels are smart carriers that sense and adapt to local pathological changes, offering distinct advantages in psoriasis (Shahi et al., 2024). Beyond traditional cues like temperature or pH, disease-specific biochemical signals provide superior spatiotemporal precision. ROS, abnormally elevated in psoriatic lesions, serve as a prime endogenous trigger (Wang et al., 2025b).

Building on the aforementioned material selections and cross-linking strategies, hydrogels with diverse stimuli-responsive functionalities can be engineered. A representative example is ROS-responsive MNs fabricated from HA-PBA/EGCG boronic esters, which enable biphasic delivery: MTX diffused immediately to suppress hyperproliferation, while H₂O₂ triggered EGCG release proportionally to lesion severity (up to 90.2% over 48 h at 0.1 mM) (Fig. 8). Released EGCG scavenged intracellular ROS and inhibited NF-κB p65 activation, downregulating TNF-α and IL-17 A. This “fast-onset + on-demand” mechanism effectively remodeled the inflammatory microenvironment. Consequently, this system outperformed conventional MNs in IMQ mice, achieving superior epidermal thinning and sustained therapeutic effects (Bi et al., 2023).

### 5.3 Enhanced penetration

Psoriatic lesions exhibit hyperkeratosis and barrier dysfunction, hindering transdermal delivery. Conventional topicals fail to penetrate the thickened epidermis and are rapidly cleared (Kang et al., 2024). High dosing is often required to maintain efficacy, yet this elevates the risk of local irritation (Zhang et al., 2022). Integrating nanocarriers with hydrogel matrices synergizes follicular penetration with cutaneous adhesion, prolonging retention and boosting therapeutic efficiency(Li et al., 2024b).

As a complement, the advanced hydrogel designs we described above are also illustrated. DESs and DES-based eutectogels similarly disturb keratin packing via hydrogen-bond donation (Datta et al., 2024), fluidizing the stratum corneum to facilitate diffusion (Islam et al., 2024). ILs disrupt intercellular lipids and fluidize the stratum corneum, increasing RNP transdermal flux ∼2.5-fold and confining delivery to the epidermis—the primary GLUT1-positive target (Yin et al., 2026).

Similarly, Car@NMs@MTX-ZA hydrogels were engineered by embedding MTX-loaded ZnO/Ag mesoporous microspheres within Poly (ε-caprolactone) -*block*-poly (ethylene glycol) -*block*-poly (ε-caprolactone) (PCL-PEG-PCL) micelles dispersed in Carbopol. Micelles traverse the disordered lipid barrier via amphiphilicity. In vivo, this system delivered detectable dermal fluorescence within 2 h, with residual drug retained at 24 h—far surpassing unmodified controls (Fig. 9). Therapeutically, it dual-targets psoriasis by inhibiting p65 phosphorylation to suppress innate inflammation (TNF-α, IL-23, IL-6) and blocking ROS-mediated STAT3/cyclin D1 activation to curb keratin 17 expression and keratinocyte hyperproliferation (Xu et al., 2022).

### 5.4 Advanced formulations

Conventional semisolid formulations are no longer the sole option for topical psoriasis therapy (Zhao et al., 2020). Hydrogels now serve as versatile matrices that can be integrated with microneedles or spray devices to overcome skin barrier limitations and enhance transdermal drug delivery (Sharma et al., 2024). This synergy between materials and dosage forms facilitates enhanced drug efficacy and balances deep permeation with local enrichment (Omidian and Dey Chowdhury, 2025). Consequently, drug delivery efficiency in lesional skin is significantly boosted (Hardy et al., 2016).

Based on black phosphorus-incorporated inverse opal hydrogels constructed via self-assembly, the microneedle system exhibits efficient photothermal conversion and structural responsiveness. Specifically, NIR irradiation triggers swelling or degradation, elevating drug release from ∼10% to over 50%, thereby conferring precise spatiotemporally controlled, on-demand release capability. (Lu et al., 2024b) (Fig. 10(a)). GelMA/PVA-based dissolvable MNs have emerged as a robust platform for psoriasis, enabling sustained release of MTX (10 d) and Pue (30 d) (Zhao et al., 2025). These hydrogels improve drug penetration more effectively (GhavamiNejad et al., 2025).

Beyond MNs, sprayable in situgels address large, irregular lesions via rapid, “zero-touch” coverage (Altunbek et al., 2024). Exemplified by LA/tea polyphenol-loaded thermosensitive liposomes, this system achieves 2.3-fold higher epidermal deposition than standard gels within 2 s. Mechanistically, it suppresses TNF-α/NF-κB/AP-1 signaling to remodel immunity, while ensuring 3-day sustained release and deep penetration, thereby alleviating psoriasis (Shen et al., 2025) (Fig. 10(b)).

## 6 Challenges and prospects

Although hydrogels have attracted much attention in the treatment of psoriasis, the inherent limitations of the drug itself are often overlooked (Clegg et al., 2024). To move from the laboratory to the clinic, there must be considerations of limited material selection, scale-up of production challenges, and regulatory approval.

Current hydrogels struggle to balance mechanics, adhesion, drug loading, and biocompatibility. Complex designs often induce cytotoxicity, immunogenicity, and instability (Hodgson et al., 2017). Cumbersome fabrication further hinders reproducibility and scalability. Overcoming these barriers demands simplified formulations, robust processes, and batch consistency for stable production (Richbourg et al., 2024).

Current hydrogels face translational hurdles in ensuring efficacy, safety, and acceptability (Liang et al., 2026). For psoriasis, unreliable drug release, poor retention, and incomplete barrier repair exacerbate this gap (Kumar et al., 2026). Formulation complexity triggers unpredictable responses and safety concerns. Addressing these issues demands clinically relevant models, standardized endpoints, and long-term data. AI-driven optimization offers a promising route to enhance clinical outcomes (Das et al., 2009; Smith et al., 2024).

Industrial translation is often hindered by poor reproducibility, limited scalability, and inadequate storage stability. Although many advanced hydrogels show promise in the lab, their scale-up remains constrained by complex processes, high costs, and inconsistent batch quality (Akbari et al., 2023). Water-rich or bioactive hydrogels are particularly problematic, suffering from dehydration, microbial contamination, and cold-chain dependence. Overcoming these barriers requires scalable manufacturing routes, storage-stable formats such as freeze-dried systems, and cost-effective designs to meet market demands (Wang and Burgess, 2013).

Regulatory and market access are hindered by ambiguous standards and reimbursement hurdles. Complex hydrogels pose safety challenges ill-suited to current frameworks (Xu et al., 2024). High production costs further undermine cost-effectiveness and adoption. Overcoming these barriers requires aligning innovation with regulatory expectations via defined chemistry, robust control, and clear benefit-risk profiles. Demonstrating clinical superiority and health-economic value is essential for successful translation (Paul et al., 2017).

## 7 Conclusion

Advanced hydrogels offer biocompatibility, controlled release, and enhanced penetration for psoriasis therapy. Despite improving drug retention and efficacy preclinically, no hydrogel-based treatment has yet achieved clinical approval. Major translational barriers include formulation stability, scalable manufacturing, and long-term safety. Future progress demands multidisciplinary integration of material design, AI-assisted optimization, and mechanistic insight, particularly regarding the microbiome–metabolic axis. With rigorous validation, advanced hydrogels may enable personalized, high-precision psoriasis therapy.

## CRediT authorship contribution statement

**Huijuan Liang:** Writing – original draft, Investigation. **Huina Cao:** Visualization, Investigation. **Shulin Pu:** Visualization, Investigation. **Runqi Xu:** Investigation. **YaQi Xu:** Investigation. **Chengxiao Wang:** Writing – review & editing, Conceptualization.

## Declaration of competing interest

The authors declare that they have no known competing financial interests or personal relationships that could have appeared to influence the work reported in this paper.

## Data Availability

Data will be made available on request.
